# Prevalence of vancomycin resistant *Enterococci* and associated risk factors among clients with and without HIV in Northwest Ethiopia: a cross-sectional study

**DOI:** 10.1186/1471-2458-14-185

**Published:** 2014-02-20

**Authors:** Wondwossen Abebe, Mengistu Endris, Moges Tiruneh, Feleke Moges

**Affiliations:** 1 Department of Medical Microbiology, University of Gondar, Gondar, Ethiopia

**Keywords:** Vancomycin-resistant, Enterococcci, HIV

## Abstract

**Background:**

*Enterococci* are the most important multidrug resistant organisms associated with immunocompromised patients. Data are lacking about the epidemiology of vancomycin resistant *Enterococci* (VRE) in Ethiopia. This study aimed to assess the prevalence of VRE, their susceptibility patterns to different antibiotics and associated risk factors in fecal samples of Human Immunodeficiency Virus (HIV) positive and HIV negative clients.

**Methods:**

A cross sectional study was carried out in a total of 226 (113 HIV positive and 113 HIV negative) clients, from July 1/2013 to September 30/2013 at the University of Gondar Teaching Hospital. Data on socio-demographic characteristics and risk factors were collected with a short interview guided by pre-tested structured questionnaire. The enterococci were isolated and identified from stool sample using standard bacteriological procedures. Kary Bauer disk diffusion method was used to determine the susceptibility patterns of *Enterococci* isolates. Data were entered and analyzed using SPSS version 20 statistical package.

**Results:**

The overall colonization of *Enterococci* was 88.9% (201/226) of which 11 (5.5%) were VRE. The prevalence of VRE among clients with and without HIV infections were 8(7.8%) and 3(3.1%), respectively. Ninety percent of the *Enterococci* isolates (181/201) were resistant to two or more antibiotics tested. Isolates of *Enterococci* recovered from stool samples of HIV infected patients were more resistant to amoxicillin and amoxicillin-calvulinic acid than HIV negative clients (P < 0.05). Antibiotic treatment for the last 2 weeks was found to be the risk factor that showed statistically significant association with the presence of high VRE colonization. However, the socio-demographic variables and factors such as malnutrition, leucopenia, thromobocytopenia, anaemia, duration of Highly Active Antiretroviral Therapy, CD4 cell count, stage of WHO and drinking alcohol were not associated with VRE (P > 0.05).

**Conclusion:**

The high prevalence of VRE in this study signals the emergence of VRE in the study area. Prior antibiotic treatment was associated with VRE colonization. Therefore, rational use of antibiotics and more detailed study using phenotypic and genotypic methods are needed.

## Background

*Enterococci* are normal inhabitants of the gastrointestinal tract of humans and animals. Two species cause most enterococcal infections, *Enterococcus faecalis* and *E. faecium. Enterococci* cause urinary tract infections mainly followed by intra-abdominal and pelvic infections. They also cause surgical wound infections, bacteraemia, endocarditis, neonatal sepsis and rarely meningitis [1].

Among the risk factors, previous antibiotic treatment [2], duration of hospitalization (≥7 days), and duration of vancomycin use (≥7 days) [3], surgical units or intensive-care units; co-morbidities such as diabetes, renal failure; and the presence of a urinary catheter [4], are among the many risk factors for colonization or infection with vancomycin resistance *Enterococci* (VRE). Critically ill patients or those with severe underlying disease or immunosuppression such as persons who have had an intra abdominal or cardio-thoracic surgical procedure or an indwelling urinary or central venous catheter are also important risk factors [5]. Moreover, HIV-positive patients often receive antibiotic therapy and have frequent contact with the healthcare system, both of which are factors that have been associated with an increased risk of infection with VRE in other populations [6].

The relative importance of *Enterococcus* as a pathogen has increased with the occurrence of high-level resistance to multiple antimicrobial drugs, such as ampicillin and vancomycin [7]. The emergence of VRE has alarmed the global infectious diseases community due to few option left for disease management, resistance gene transfer from *Enterococci* to *Staphylococcus aureus* and presence of different selection pressures for VRE proliferation and rapid expansion of resistant populations [8]. The prevalence of VRE was reported in Europe, Asia, Australia, South America and some countries of Africa [9]. However, there is no enough data available on the epidemiology and risk factors of VRE in Ethiopia. The present study aimed to assess the prevalence of VRE, their susceptibility patterns to different antibiotics and associated risk factors in fecal samples of HIV positive and HIV negative clients at the University of Gondar Teaching Hospital.

## Methods

### Study design, area and period

A cross sectional study was carried out from July 1/2013 to September 30/2013 at the University of Gondar Teaching Hospital. The hospital is located in Gondar town, 737 km Northwest of Addis Ababa. It has 400 beds and provides health care referral services for over 5 million inhabitants in Northwest Ethiopia. The University Teaching hospital has ART clinic and blood bank center.

### Study population

HIV positive patients attending ART clinic and HIV negative blood donors at the University of Gondar Teaching Hospital during the study period were enrolled in this study.

### Sample size and sampling technique

The sample size was determined by using EPI INFO version 3.5.3 in double population proportion. Since there is no previous study done in this area, 50% prevalence was used for HIV negative clients. HIV positive patients are assumed to have 20% more VRE carriage than HIV negative clients, 70%. Considering the two prevalence, power 80% at 95% confidence with 1:1 ratio the final sample size was 226 (113 HIV infected patients and 113 HIV negative blood donors). The last 3 years hospital report showed that an average of 57 HIV positive clients was visited the ART laboratory daily. We calculated the total participants in 3 months study period (57 × 90 days = 5130). Since our sample size is 113 HIV positive patients we calculated the k = value to be 45. The first one was selected with lottery method from 1-45 cases. Then every 45^th^ patients were included in this study. Consecutive HIV negative volunteers blood donors (n = 113) were also included in the study.

### Data collection

Socio-demographic and clinical data and other possible factors responsible for colonization of VRE such as antimicrobial therapy, drinking alcohol and others were collected using pre-tested structured questionnaire. Five milliliters of blood was drawn from each client by an experienced laboratory technologist. The HIV status of the clients was determined using rapid test kit (KHB, STAT pak and Uni-Gold) following the current Ethiopian algorithm [10]. The CD_4_ count (for HIV positives) and complete blood count were done using BD FACS count and Sysmex Machine respectively.

### Stool sample collection and processing

The clients were provided with wide-mouthed, sterile plastic containers and informed to submit stool specimens. The collected stool specimens were streaked on Bile Esculin Azide agar (Hardy Diagnostics, Santa Maria, CA, USA) and incubated for 24 hours at 37°C. Plates were observed for appearance of characteristic colonies of growth and blackening. Typical characteristic colonies were selected randomly for characterization and presumptive identification of *Enterococcci*[11]. Each isolate was also assessed using Gram staining, catalase test and its growth in 6.5% NaCl broth. Presumptive pure colonies were picked and inoculated into Brain Heart Infusion (BHI) broth, and incubated at 45°C for 24 hours and growth in the medium was assessed by its turbidity. An isolate fulfilling the above criteria was assumed to be *Enterococccus* species [11].

### Antimicrobial susceptibility testing

Antimicrobial susceptibility testing for each isolate was done using Muller-Hinton agar (OXOID, UK) disc diffusion method [12]. Antimicrobial susceptibility patterns of *Enterococci* were assessed against nine antibiotic discs (all OXOID, UK). These were vancomycin 30 μg, ampicillin 10 μg, amoxicillin 10 μg, amoxicillin-clavulinic acid 10 μg, ciprofloxacin 5 μg, chloramphenicol 30 μg, erythromycin 15 μg, gentamicin 10 μg and trimethoprim-sulfamethoxazole 1.25/23.75 μg.

### Quality control

Reference strain of *Staphylococcus aureus* ATCC 25923 and *E. faecalis* ATCC 51299 were used as negative and positive control, respectively.

### Data analysis

Data were entered and analyzed using SPSS version 20. Descriptive statistics were employed and odds ratio (OR) was used to determine the strength of association. Multivariate analysis using logistic regression model was used to analyze the association between VRE colonization and potential risk factors. Those variables with over all P value less than 0.2 in the bivariate analysis were entered into multivariate analysis. P-values < 0.05 was considered statistically significant.

*Definitions:* Leukocytosis and leucopenia were defined as having WBC count greater than 12,000/mm3 and less than 4,000/mm3, respectively. A client was named as thrombocytopenic when she/he had a platelet count of <100,000/ μL.

### Ethical consideration

The study was approved by the research and ethics committee of the School of Biomedical and Laboratory Sciences, University of Gondar. Written informed consent was also obtained from each client.

## Results

### Socio-demographic characteristics of the clients

A total of 226 (113 HIV positive and113 HIV negative) clients were included in this study. One hundred and twenty three (54.4%) were males and 103 (45.6%) were females. The mean age and standard deviation was 35.62 ± 9.54 ranged from 19-66 years. Majority of the clients were literate 174 (77.0%) and urban residents 213(94.2%) (Table 1).

**Table 1:** Socio-demographic characteristics of clients with and without HIV at the University of Gondar Teaching Hospital, Northwest Ethiopia, 2013

**Socio-demographic characteristics**	**HIV status**		**All (n = 226)**
	**Negative (n = 113)**	**Positive (n = 113)**	
	**Number (%)**	**Number (%)**	**Number (%)**
Sex			
Male	70 (61.9)	53 (46.9)	123 (54.4)
Female	43 (38.1)	60 (53.1)	103 (45.6)
Mean (SD) Age (range)	35.12 ± 10.85 (19-66)	36.12 ± 8.05 (23-65)	35.62 ± 9.54 (19-66)
Age (years)			
11-20	7 (6.2)	0 (0)	7 (3.1)
21-30	39 (34.5)	35 (31.1)	74 (32.7)
31-40	43 (38.1)	53 (46.9)	96 (42.5)
41-50	15 (13.3)	21 (18.6)	36 (15.9)
51-60	6 (5.3)	3 (2.7)	9 (4.0)
61+	3 (2.1)	1 (0.9)	4 (1.8)
Education status			
Illiterate	21 (18.6)	31 (27.4)	52 (23.0)
Literate	92 (81.4)	82 (72.6)	174 (77.0)
Residence			
Urban	103 (91.2)	110 (97.3)	213 (94.2)
Rural	10 (8.8)	3 (2.7)	13 (5.8)
Marital status			
Single	46 (40.7)	23 (20.4)	69 (30.5)
Married	64 (56.6)	51 (45.1)	115 (50.9)
Widowed	2 (1.8)	14 (12.4)	16 (11.5)
Divorced	1 (0.9)	25 (22.1)	26 (7.1)

### Prevalence of VRE among HIV positive and HIV negative clients

Among 226 clients growth of *Enterococcus* species was seen in 201 (88.9%) stool specimens. The colonization of *Enterococci* among HIV positives and HIV negatives clients was 103 (91.2%) and 98 (86.7%), respectively. There was no statistically significant difference in the isolation frequency of *Enterococci* among clients with and without HIV infection (P = 0.29).

Among the 201 isolates of *Enterococci* in this study, 11(5.5%) were VRE. The prevalence of VRE among HIV positive and HIV negative clients was 8 (7.8%) and 3 (3.1%), respectively. No significant association was observed between VRE and HIV sero-status (P = 0.14; OR = 2.67; 95% CI = 0.62– 13.12) (Table 2).

**Table 2:** Prevalence of VRE among clients with and without HIV at the University of Gondar Teaching Hospital, Northwest Ethiopia, 2013

**HIV status**	**Total**	***Enterococci***		**OR (95% CI)**	**P-value**
		**VRE**	**VSE**		
		**Number (%)**	**Number (%)**		
Positive	103(51.2)	8(7.8)	95(92.2)	2.67(0.62 – 13.12)	0.14
Negative	98(48.8)	3(3.1)	95(96.9)	1.00	
Total	201(100)	11(5.5)	190(94.5)		

VRE = Vancomycin resistant *Enterococci*; VSE = Vancomycin Sensitive *Enterococci*.

### Antimicrobial susceptibility pattern of Enterococcci

One hundred eighty one (90%) isolates were resistant to two or more antimicrobials tested. Among 201 *Enterococci* isolates tested 113 (56.2%) were resistant to amoxicillin. The amoxicillin resistance *Enterococci* were 65(63.1%) and 48 (42.5%) among HIV positives and HIV negative clients, respectively. Fifty five isolates (27.4%) of *Enterococci* were resistant to amoxacillin-clavulinic acid, of which, 35 (33.9%) and 20 (20.4%) were from HIV positives and HIV negatives, respectively. There was statistically significant difference of resistance to amoxicillin, amoxacillin-clavulinic acid and erythromycine between isolates from HIV positive and HIV negative clients (P < 0.05). However, no statistically significant differences were observed in resistance pattern for the antibiotics: ampicillin, chloroamphinicol, ciprofloxacillin, gentamycine, sulphamethaxozole and vancomycin in HIV positives and HIV negative clients (P > 0.05) (Table 3).

**Table 3:** Antimicrobial resistance patterns among clients with and without HIV at the University of Gondar Teaching Hospital, Northwest Ethiopia, 2013

**Resistant to antibiotics**	**HIV positive N = 103**	**HIV Negative N = 98**	**Total N = 201**	**X**^**2**^	**P-value**
	**Number (%)**	**Number (%)**	**Number (%)**		
Ampicillin	80(77.7)	80(81.6)	160(79.6)	0.27	0.49
Amoxacillin	65(63.1)	48(48.9)	113(56.2)	4.07	0.04
Amoxacillin-clavulinic acid	35(33.9)	20(20.4)	55(27.4)	4.65	0.03
Chloroamphinicol	13(12.6)	12(12.4)	25(12.4)	0.01	0.94
Ciprofloxacillin	41(39.8)	27(27.5)	68(33.8)	3.37	0.06
Erythromycine	58(56.3)	69(70.4)	127(63.2)	4.29	0.07
Gentamycine	73(70.8)	71(72.4)	144(71.6)	0.06	0.80
Sulphamethaxazole	45(43.6)	48(48.9)	93(46.4)	0.57	0.45
Vancomycine	8(7.7)	3(3.1)	11(5.5)	2.15	0.14
MDR	91(92.8)	90(91.8)	181(90)	0.68	0.41

All isolates of VRE were multidrug resistant and all isolates of VRE in HIV positive patients were resistant to Ampicillin, Amoxacillin and Gentamycin while in HIV negative patients all isolates of VRE were resistant to Ampicillin, Erythromycin and Trimethoprim-sulfamethoxazole (Table 4).

**Table 4:** VRE (n = 11) isolates resistant patterns among clients with and without HIV at the University of Gondar Teaching Hospital, Northwest Ethiopia, 2013

**Resistant to antibiotics**	**HIV positive N = 8**	**HIV Negative N = 3**	**Total N = 11**
	**Number (%)**	**Number (%)**	**Number (%)**
Ampicillin	8 (100)	3 (100)	11 (100)
Amoxacillin	8 (100)	1 (33.3)	9 (81.8)
Amoxacillin-clavulinic acid	5 (62.5)	1 (33.3)	6 (54.5)
Chloroamphinicol	3 (37.5)	2 (66.7)	5 (45.5)
Ciprofloxacillin	2 (25)	2 (66.7)	4 (36.4)
Erythromycin	7 (87.5)	3 (100)	10 (90.9)
Gentamycin	8 (100)	2 (66.7)	10 (90.9)
Trimethoprim-sulfamethoxazole	4 (50)	3 (100)	7 (63.6)
MDR	8 (100)	3 (100)	11 (100)

### Associated risk factors with VRE

Majority of VRE was isolated from females, age > 31 years and urban residents. No significant association between VRE and socio-demographic characteristics such as, age, sex, educational status, marital status and residence (P > 0.05) (Table 5).

**Table 5:** Socio-demographic characterstics and prevalence of VRE with and without HIV at the University of Gondar Teaching Hospital, Northwest Ethiopia, 2013

**Socio-demographic characteristic**	**VRE (n = 11)**		**P-value**	**OR (95% CI)**
	**HIV positive**	**HIV negative**		
	**No. (%)**	**No. (%)**		
Sex				
Male	3 (60.0)	2 (40.0)	---	1.00
Female	5 (83.3)	1 (16.7)	0.54	3.33 (0.12-156)
Age (years)				
11-20	0 (0)	0 (0)	NA	NA
21-30	2 (100)	0 (0)	---	1.00
31-40	4 (80.0)	1 (20.0)	0.71	0.00 (0-76.65)
41-50	2 (50.0)	2 (50.0)	0.40	0.00 (0-14.83)
51-60	0 (0)	0 (0)	NA	NA
61+	0 (0)	0 (0)	NA	NA
Education status				
Illiterate	1 (33.3)	2 (66.7)	0.15	0.07 (0.00-2.88)
Literate	7 (87.5)	1 (12.5)	---	1.00
Residence				
Urban	7 (70.0)	3 (30.0)	---	1.00
Rural	1 (100)	0 (0)	0.73	UD
Marital status				
Single	1 (100)	0 (0)	---	1.00
Married	4 (57.1)	3 (42.9)	0.62	0.00 (0-40.29)
Widowed	2 (100)	0 (0)	NA	NA
Divorced	1 (100)	0 (0)	NA	NA
**Over all**	**8 (72.7)**	**3 (27.3)**	**0.14**	**2.67 (0.62-13.12)**

Prevalence of VRE was significantly associated with antibiotic treatment more than 2 weeks (P = 0.01; AOR = 10.21; 95% CI = 1.31–217.40). However, it was not associated with HAART, duration of HAART, CD_4_ cell count, WBC counts, platelet counts, nutritional status, WHO stage, drinking alcohol habit and anemic status (Table 6).

**Table 6:** Risk factors of VRE among HIV positive and HIV negative clients at Gondar University Teaching Hospital, Northwest Ethiopia, 2013

**Risk factors**	***Enterococci***		**Total Number (%)**	**P-value**	**AOR (95% CI)**
	**VRE**	**VSE**			
	**Number (%)**	**Number (%)**			
Antibiotic treatment (>2 weeks)					
Yes	10 (9.6)	94 (90.4)	104 (51.7)	0.01	10.21 (1.31 – 217.40)
No	1 (1.0)	96 (99.0)	97 (48.3)	---	1.00
CD_4_^*^					
≤350	5 (11.4)	39 (88.6)	44 (42.7)	0.21	2.39 (0.46 – 13.57)
>350	3 (5.1)	56 (94.9)	59 (57.3)	---	1.00
HAART					
Yes^*^	8 (7.8)	94 (92.2)	102 (50.7)	0.13	2.72 (0.63 – 13.40)
No	3 (3.0)	96 (97.0)	99 (49.3)	---	1.00
HAART Duration (Year)^*^					
≤2	2 (6.7)	28 (93.3)	30 (29.1)	---	1.00
>2	6 (8.2)	67 (91.8)	73 (70.9)	0.57	1.25 (0.21 – 9.62)
WBC counts					
Normal	11 (6.6)	156 (93.4)	167 (83.1)	---	1.00
Leucopenic	0 (0)	27 (100)	27 (13.4)	0.18	0.00 (0.00 – 2.92)
Leucocytosis	0 (0)	7 (100)	7 (3.5)	0.62	0.00 (0.00 – 12.95)
Platelet					
Normal	10 (5.6)	168 (94.4)	188 (88.6)	---	1.00
Thrombocytopenic	0 (0)	12 (100)	12 (6.0)	0.51	0.00 (0.00 – 8.40)
Thrombocytosis	1 (9.1)	10 (90.9)	11 (5.4)	0.49	1.68 (NA)
Nutritional status					
Normal	8 (4.7)	161 (95.3)	169 (84.1)	---	1.00
Malnourished	2 (13.3)	13 (86.7)	15 (7.5)	0.19	3.10 (0.41 – 18.57)
Overweight	1 (5.9)	16 (94.1)	17 (8.5)	0.59	1.26 (NA)
Anemic					
Yes	11 (5.8)	179 (94.2)	190 (94.5)	0.52	UD
No	0 (0)	11 (100)	11 (5.5)	---	1.00
Drinking alcohol					
Yes	2 (3.4)	57 (96.6)	59 (29.4)	0.32	0.52 (0.07 – 2.69)
No	9 (6.3)	133 (93.7)	142 (70.6)	---	1.00
WHO stage^*^					
I	2 (8.3)	22 (91.7)	24 (23.3)	---	1.00
II	0 (0)	15 (100)	15 (14.6)	0.37	0.00 (0.00 – 6.91)
III	2 (6.7)	28 (93.3)	30 (29.1)	0.60	0.79 (0.07 – 8.68)
IV	4 (11.8)	30 (88.2)	34 (33.3)	0.51	1.470.20 – 12.82)

^*^Only for HIV infected patients.

## Discussion

In the present study the overall prevalence of *Enterococci* was 201/226(88.9%). This was consistent with the previous report from Israel 88.5% [13]. However, it was lower than a report from Algeria (100%) [14]. This variation in colonization might be due to the advanced molecular method in the previous study.

The prevalence of *Enterococci* was higher in HIV positives (91.2%) than HIV negatives (86.7%) clients. However, this was not statistically significant (P = 0.14).This result was in line with reports by Hijazi [15] in hospitalized patients (94%) and in individuals living in the community (89%).

The prevalence of VRE in this study was 11/201(5.5%). This was consistent with reports from Korea, 4.5% [16] and lower than reports from Egypt (25%) isolated from pediatric patients [17] and health care workers in intensive care units (9.5%) [18]. These differences might be due to the study design and study participants (pediatric age and intensive care unit) in the previous study.

The prevalence of VRE among HIV positives (7.8%) in the present study was higher than reports from USA, 0% [19] and 4.7% [6]. This variation might be due to difference in the study population where most of the clients in the present study had habit of animal contact and this was supported by Bekele and Ashenafi, reported as 100% VRE *Enterococci* isolated from faeces of chicken and cattle in Ethiopia [20]. Comparison of colonization of VRE among HIV positives (7.8%) and HIV negative (3.1%) clients’ revealed that there was a slightly higher VRE in HIV positive patients. The higher VRE among HIV positives might be due to repeated exposure to different antibiotics [6] and immune-suppression [21] which are important risk factors for colonization or infection with VRE.

Among risk factors studied for VRE, a patient with a history of prior antibiotic use was found to be important factor (P = 0.01). Similar reports from Iran showed that antibiotic exposure can cause the emergence of VRE by inducing the expression of resistance genes and by selecting strains already expressing these genes and altering the competing microbial flora in the GI tract, thereby increasing VRE concentration in the stools [22,23]. However, stool consistency, CD4+ count, HARRT, HARRT duration (year),WBC and platelet count, nutritional status, anaemia, drinking alcohol, WHO stage for HIV were not statistically significant risk factors for VRE (P > 0.05). This was consistent with previous study in USA [6].

Among 201 isolates of *Enterococci* 181 (90%) were resistant to two or more antibiotics tested. Similarly over 80% of multiple drug resistance (MDR) *Enterococci* isolates were reported from cattle in Addis Ababa [20]. Although high percentage of resistance against ampicillin (79.6), gentamaycin (71.6) and erythromycin (63.2) were observed, no significant difference was seen between HIV positive and HIV negative clients. However, the resistance pattern of *Enterococcus* isolates to amoxicillin and amoxicillin-clavulinic acid were significantly higher in HIV positive than HIV negative clients. This significant difference might be due to the frequent prescription of broad spectrum antibiotic for HIV patients. The high prevalence of MDR enterococccal colonization in humans may lead to infection with reduced treatment options.

### Limitation of the study

The isolated enterococcci were not indentified to species level.

## Conclusion

The prevalence of VRE in clients attending the University of Gondar Hospital was 5.5%. Patients with prior exposure to antibiotics for more than two weeks had higher colonization than their respective group. The presence of 11 (5.5%) prevalence of VRE in this study signals the emergence of VRE in the study area. Therefore, rational use of antibiotics and more detailed study using phenotypic and genotypic methods are needed.

## Competing interests

The authors declare no conflict of interest exists.

## Authors’ contributions

WA, ME and FM: conceived the idea, write the proposal, processes clinical samples, data analysis and draft the manuscript for publication. MT: assisted during sample processing. All the authors read and approved the manuscript.

## Pre-publication history

The pre-publication history for this paper can be accessed here:

http://www.biomedcentral.com/1471-2458/14/185/prepub
